# The Efficiency of Live-Capture Traps for the Study of Red Fox (*Vulpes vulpes*) Cubs: A Three-Year Study in Poland

**DOI:** 10.3390/ani10030374

**Published:** 2020-02-26

**Authors:** Ewa J. Mierzejewska, Dorota Dwużnik, Katarzyna Tołkacz, Anna Bajer, Marek Panek, Maciej Grzybek

**Affiliations:** 1 Department of Eco-Epidemiology of Parasitic Diseases, Institute of Developmental Biology and Biomedical Sciences, Faculty of Biology, University of Warsaw, 00-927 Warsaw, Poland; dorota.dwuznik@biol.uw.edu.pl (D.D.); k.h.tolkacz@gmail.com (K.T.); anabena@biol.uw.edu.pl (A.B.); 2 Polish Hunting Association, Research Station, Sokolnicza 12, 62-055 Czempiń, Poland; m.panek@pzlow.pl; 3 Department of Tropical Parasitology, Institute of Maritime and Tropical Medicine, Medical University of Gdańsk, 80-210 Gdańsk, Poland; grzybek.genetics@gmail.com

**Keywords:** wild canids, trapping success, trap rate, trapping methodology, weather

## Abstract

**Simple Summary:**

Wild canids may be captured for a variety of purposes, including, for example, scientific research, conservation or relocation to new sites. The use of conventional live-capture traps (also referred to as cage traps), which involve safe trapping techniques, may be limited by the age of the target animal, landscape structure, proximity to areas intensively used by people and regulations at the national or regional level. Live-trapping with cage traps is the only legal method for capturing juvenile red foxes in Poland. However, little is known about the factors that can affect capture efficiency of these trapping devices. In this research, we developed standard operating procedures (SOPs) for the capture of fox cubs using cage traps and camera traps. We also identified factors that may facilitate or hamper the success of cage traps. The selectivity (total number of trapped red fox cubs per total number of trapped animals) of live-capture traps (91.4%) and the probability of capturing one cub per night (70.2%) were high. In the vicinity of human settlements, fewer cubs explored and entered cage traps. Capture efficiency was also affected by the weather, i.e., it increased in poor weather conditions (e.g., during rain, thunderstorms or ground frost). None of the trapped animals were injured. Live-trapping with cage traps can be an effective and safe alternative to other devices used to trap juvenile foxes. However, capture-efficiency is affected by trappers’ experience and a range of factors including weather conditions and distance to human settlements.

**Abstract:**

Safe and efficient techniques for the live capture of carnivores are limited. In this study, we identified some of the factors that could affect the success of capturing red fox cubs with live capture traps (also known as cage traps). During a three-year period, we analysed 32 captures of 25 fox cubs (1.3 captures/fox). We assessed the impact of the following factors: sex of animals, month of trapping, weather conditions recorded for each trap-night, the willingness of cubs to explore and enter cage traps, the researchers’ activity around den complexes before trapping and distances to the nearest village or farm. The overall trap rate (32 captures, including recaptured cubs) and the trap rate for individual cubs (25 captures) was 11.2 cubs/100 trap-nights and 8.7/100 trap-nights, respectively. Animals other than foxes were captured only three times, thus the selectivity of the cage-trapping method was high (32/35 = 91.4%). The probability of capturing one cub per night was 70.2% (32 cubs/47 nights). Cubs inhabiting dens in the vicinity of human settlements were less likely to explore and enter traps. Vixens were more likely to relocate their litters if the activity of the staff setting the traps was intense at the trapping site. The success of trapping was higher during poor weather as, for example, during rain or thunderstorms. None of the trapped animals suffered any injuries. Whereas cage trapping can be an effective and safe capture method for juvenile foxes, capture efficiency is affected by the experience of the trappers and a range of other factors including weather and distance to human settlements.

## 1. Introduction

Wild carnivores may be live-captured for a variety of purposes, i.e., conservation of endangered species, behavioural studies, removal from residential areas, pest control or surveillance of zoonotic diseases [1,2,3]. Live-capturing makes it possible to collect fresh material more than once from known individuals, install radio-collars or tags, and to follow the life history of particular animals [4,5,6].

Wild carnivores can be captured in foothold traps, leg snares, cage traps, or modified neck snares with safety stops [2,6,7,8]. The use of these devices may be limited by the size or age of the animals, landscape characteristics, proximity to areas intensively used by people, and regulations at national or regional levels [7]. Cage/box traps and restraining snares have been identified as safe methods for trapping juvenile fox [4,6]. Utilization of snares with a safety stop has been shown to be a very efficient tool [6], however, use of this device is prohibited by law in some countries, including Poland. As noted by Baker et al. [4], empirical data on the efficacy of different types of traps utilized for capture of carnivores are still limited. 

In the course of a three-year study focusing on the prevalence of tick-borne diseases in selected populations of red foxes (*Vulpes vulpes*) in Poland, cubs were live-captured for blood sampling and collection of ectoparasites. We developed efficient procedures for identifying complexes, monitoring cub activity, and trapping young animals with live-capture cage traps. Based on our observations and experience gained during the field work, we hypothesized that (i) cubs reared in close locations to human settlements are more likely to avoid traps, but (ii) poor weather conditions and previous experience of the people involved in the trappings may have a positive effect on trapping success. In this paper, we assess the efficiency of our protocols, and evaluate the influence of factors potentially hampering trapping success: staff experience, willingness of cubs to explore and enter the trap, weather conditions and distance to the closest human settlements.

## 2. Material and Methods

### 2.1. Study Area

**Figure 1:**
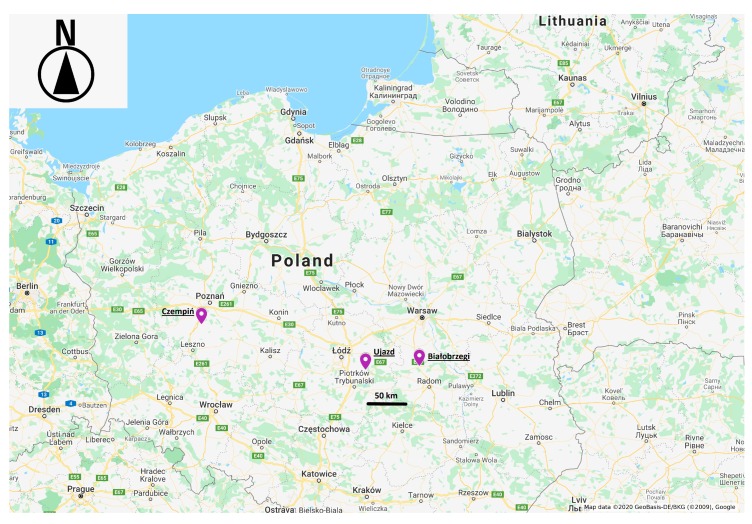
The three regions where the red fox cubs were trapped are indicated on the contour map of Poland (Google Maps, 2020) [9].

Trapping was conducted in three regions of Poland: in the surroundings of Białobrzegi in the Mazowieckie Voivodeship (N 51.6460, E 20.9566), in Ujazd in the łódzkie Voivodeship (N 51.5995, E 19.9212) and in Czempiń in the Wielkopolskie Voivodeship (N 52.1427, E 16.7612) (Figure 1). Trapping was conducted during the breeding season in May and June in 2016–2018. During these months in Poland, cubs usually start to explore the environment outside their dens [8]. Consent for the field research was issued by the First Warsaw Local Ethics Committee for Animal Experimentation in Poland (ethical license numbers 706/2015 issued on 23 April 2015 and 515/2018 issued on 30 January 2018) and consent was obtained also from the Polish Ministry of the Environment (DLP-VIII.6713.4.2016.ABR) and issued on 7 April 2016.

Białobrzegi is located in the valley of the Pilica River. The trapping area (65 km^2^) was characterised by a rolling landscape, consisting of a mosaic of coniferous or mixed forests, and agricultural areas dominated by pastures, meadows and wasteland. Trapping occurred here in May and June 2016–2018. The second site comprised the surroundings of Ujazd where we trapped in 2017 and 2018, across an area of nearly 35 km^2^. About 70% of this site is covered by coniferous forest and the remaining areas are mainly arable fields. The third study site is located around Czempiń (68 km^2^), predominantly within the territory of the General Dezydery Chłapowski Landscape Park in western Poland. This territory is mainly covered by arable land (about 15% of forest), but the landscape is enriched by mid-field tree islands, windbreaks and tree alleys along roads. Fox dens were often found here on the slopes of numerous mid-field ditches. Foxes were trapped in this area between 2017 and 2018. In each of the three study sites the closest human settlements were small villages or farms.

Den complexes were located based on data provided by local foresters and hunters, and were visited between April and May of each year. The remains of prey, tracks and scats typical of fox cubs around the dens, indicated they were inhabited. Additionally, each entrance to a den was checked for the presence of scent characteristic of foxes. On approaching seven of the den complexes, we were able to observe cubs playing. At three sites, we heard vocalization of cubs (barking and growling) from inside of the den.

When possible, the presence of cubs was confirmed and the size of the litter determined via recordings from camera traps (Bushnell Trophy Cam HD Wireless, Overland Park, KS, USA). In several cases, camera traps could not be used, because dense vegetation covered the field of view and constantly triggered recordings. Some den complexes were located in areas of frequent human activity and therefore, camera traps were not installed here due to the high risk of theft or damage. When camera traps were used, they were installed at the same time as the traps were set to keep visits to the den as low as possible. Initially traps were left open and the trigger mechanism was permanently blocked so that the animals could not activate it by entering the trap. Sites were then revisited after 4–7 days in order to evaluate the activity of foxes.

### 2.2. Types of Traps

**Figure 2:**
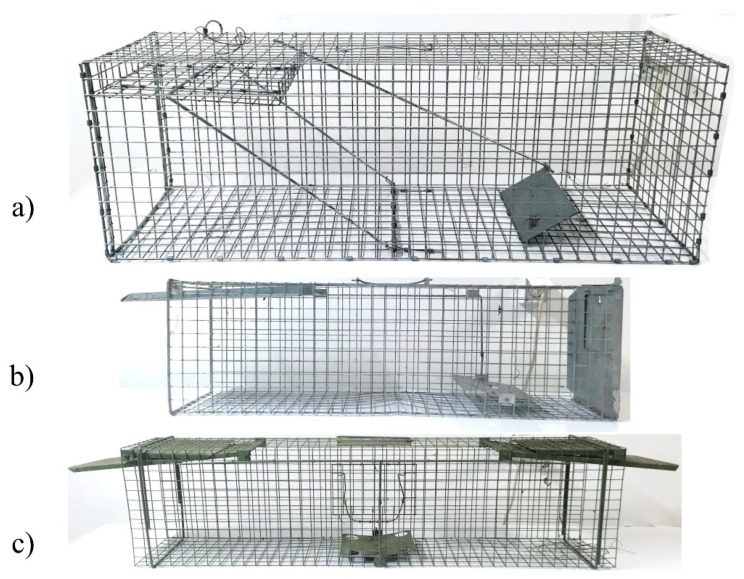
Types of live cage traps used in the study: (**a**) custom-made trap with internal wire securing the door; (**b**) trap with external wire securing the door and one opening (“Zielonołapka”, Poland); (**c**) the tunnel-like trap with external wire securing the door and two openings (”Zielonołapka”, Poland).

Twelve traps with two different types of trigger mechanism were used: the first type was custom-made for the purpose of this study with an internal wire securing the door, whereas the second type was a commercially available trap (“Zielonołapka”, Poland) with an external wire securing the door (Figure 2). Four traps of the first type (Figure 2a) were of the same size (100 × 40 × 40 cm) with one opening/door. The biggest trap of the second type (145 × 31 × 36 cm) had one opening as well as three smaller ones (80 × 21 × 31 cm) (Figure 2b). The last four traps of the second type had two openings/doors (Figure 2c) and were of the same size (110 × 21 × 31 cm). Observations during the first trapping season in 2016 showed clearly that cubs avoided entering the traps with two openings, which formed a kind of a tunnel. Thus, in the next two seasons one door in this type of traps was permanently closed.

### 2.3. Setting of the Live Cage Traps

**Figure 3:**
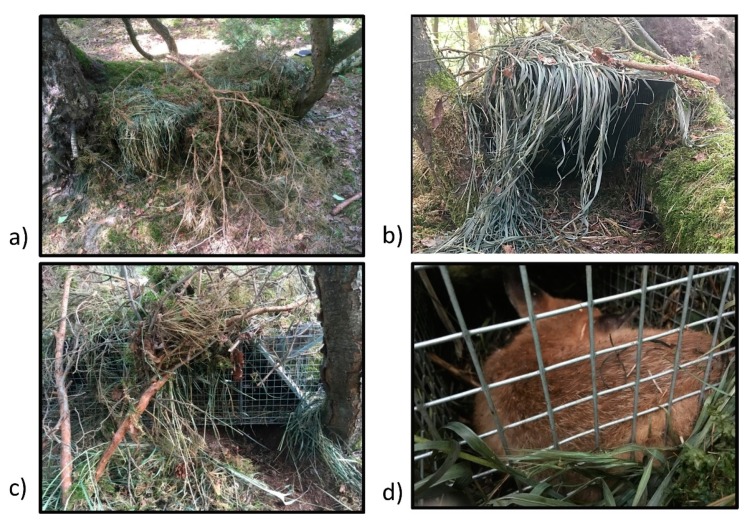
Live-capture cage trap set at the trapping site: (**a**) side view; (**b**) front view of a camouflaged trap; (**c**) the trap cover disturbed by cubs after attempts to reach the bait without entering inside; (**d**) trapped red fox cub inside the cage.

We selected inhabited den complexes with several exits (more than two). Between two and four traps were set about 10–20 m away from each den complex. The availability of places in which the traps could be well hidden was the main factor determining the number of traps set at a given site. Preferably traps were placed next to felled trees for extra stability or between bushes for cover. The floor of traps was covered by a thick layer of debris collected in situ. Traps were camouflaged by a dense cover of cut vegetation that also protected the trap from rain (Figure 3a,b). Chicken carcasses and chopped pork bones were used as bait. Pieces of the bait were hung at the back of a cage-trap and laid also at its entrance. Bait was also scattered around den sites and between den entrances and the traps. Traps were re-baited at every visit. All the equipment required for setting a trap, including the bait and vegetation used as trap coverage, was fully prepared before approaching the trap site. Setting a single trap at a site took approximately 15–25 min depending on local difficulties (i.e., terrain structure, density of vegetation). Once traps were set, any further rearrangements at the site such as adding extra traps, exchanging or replacing traps, were avoided. The decision to activate the trigger mechanism was made only after entry of cubs into a cage-trap with a blocked trigger mechanism had been confirmed. Confirmation of entry was based on recordings from camera traps or indications that bait from the back of a trap had been taken (if camera traps were not installed). If entry could not be confirmed, the traps were visited again after 3–4 days. Trapping was discontinued when cubs stopped entering the traps and/or tried to reach the bait by digging and overturning traps (Figure 3c). In such cases, the bait from the back of the trap was undisturbed and intact, but the presence of cubs was evident in the daily recordings from camera traps and/or appearance of fresh scats or tracks.

### 2.4. Trappings of Red Fox Cubs and Safety Measures

The experience gained during this 3-year study showed that even the most unexpected factors could generate unrest in the vixen, which then moved the whole litter of cubs to a different locality. Therefore, we introduced strict operating procedures (“standard operating procedures” (SOPs)) that significantly reduced disturbance in the vicinity of burrow complexes. No more than two, and always the same persons, visited a particular site. Any intensive and unnatural scents (cosmetics, cleaners, disinfectants, repellents, etc.) or odours from domestic animals (cats or dogs) were strictly avoided. As far as possible, each person used the same set of outer clothing and work gloves at each site. On the way to the den complex, the surrounding area was carefully checked for the presence of vixens or cubs. Sites were visited when the activity of animals was low as assessed by recordings from camera traps. Traps were baited and set for trapping (trigger mechanism was left unblocked) between 3:00–6:00 pm and then inspected and blocked at dawn (4:00–5:00 a.m.). The correct functioning of the trigger mechanism was always carefully inspected. Trapped red fox cubs (Figure 3d) were anesthetized prior to retrieval from the live-cage traps with a combination of ketamine (2.5 mg/kg), medetomidine (0.05 mg/kg) and butorphanol (0.25 mg/kg). After a basic clinical examination, collection of ectoparasites, a blood sample and body measurements, the animal was marked (by a shaved spot on a forehead) and when fully awake released in close proximity to the den complex. 

### 2.5. Assessment of Efficiency of Used Methodology

To assess the efficiency of the methodology used in this study, three indicators were calculated: (1) trap rate: mean number of red fox cub per 100 trap-nights (trap-night = 1 functional trap for 1 night), (2) selectivity: total number of trapped red fox cubs per total number of trapped animals and (3) the probability of capturing one cub per night: total number of trapped red fox cubs per total number of nights during which trappings were conducted.

### 2.6. Statistical Analysis

Statistical analysis was performed in order to evaluate associations between the trap rate and willingness of cubs to explore and/or enter the trap, and three additional factors: distance of the den to human settlements (six classes designated every 150 m from the nearest village or farm), weather events (rain, thunderstorm or ground frost) and the number of approaches by team members to the den complex before commencement of actual trapping (details below). The willingness of cubs to explore and/or enter the trap was assessed based on the presence of typical tracks of fox cubs around the cage-trap, eaten bait from the trap, or recordings from the camera traps (the cub entered the trap completely or at least stepped inside the cage with its front legs). In order to evaluate the significance of explanatory variables, the number of trap-nights (1 functional trap for 1 night) was associated with each category of these variables.

Statistical analysis was performed using the maximum likelihood techniques based on log linear analysis of contingency tables in the software package SPSS (version 21, SPSS, Inc., Chicago, IL, USA). The methodology used in these analyses is described in detail elsewhere [10,11]. In brief, factors incorporated in the analysis are fitted initially to all possible combinations. The analysis starts with the most complex model involving all possible main effects and interactions. Combinations that did not contribute significantly to explaining variation in the data were eliminated in a stepwise fashion beginning with the highest level of interaction (backward selection procedure). As a result, the minimum sufficient model thus obtained, for which the likelihood ratio of χ^2^ is not significant (*p* > 0.05), is considered to be adequate in explaining the data. The importance of each term in the final model is assessed by the probability that its exclusion would alter the model significantly. In the present study, four variables represented factors of interest: distance of the den to human settlements is categorized as distance (6 classes, each class covering 150 m of distance). Weather represented two categories (good or poor: rain, thunderstorm or ground frost). Human activity around the den complex before the actual trapping is described as baiting (2 levels, short ≤ 2 visits; long ≥ 3 visits). The willingness to explore and enter the trap by the cubs is defined as eagerness (2 levels, no activity inside the trap or activity confirmed). The trapped red fox cub is defined as trapped (2 levels, yes or no). Two full factorial models were constructed, which best explained the relationship between the variables: (1) distance, eagerness, trapped and weather; (2) distance, eagerness, trapped and baiting. The Chi-square test was used to evaluate the effect of month (2 levels; May and June) on (i) rate and (ii) sex of trapped cubs.

## 3. Results

During the three years of the study, 97 den complexes were inspected (Table 1). A similar number of dens were available for assessment around Czempiń (n = 40) and Białobrzegi (n = 39). In the vicinity of Ujazd, the number of known dens was lower (n = 18). In total, 53 (54.6%) inspected dens were inhabited by burrow dwelling animals. Presence of red fox cubs was detected in 41 den complexes (42.3%), including 20 (20.5%) sites where cubs were recorded by camera traps. Tracks, scats and scent characteristic for fox cubs were recognized around 21 (21.6%) of the dens where camera traps were not set. At eight sites, trapping was not undertaken, because dens were located inside fenced orchards (n = 3) or the presence of fox tracks, scats and scent were irregular (n = 2). Litters were relocated by vixens at three sites after the cage traps and camera traps had been installed. Ultimately, trapping was performed at 33 sites. European badgers (*Meles meles*) exploring dens were recorded by camera traps at 11 (11.3%) den complexes. In the remaining six (6.2%) dens, the presence of these animals was confirmed based on occurrence of fresh scats and signs of intensive activity. At two sites in the Białobrzegi area and three in the Czempiń area, den complexes were co-inhabited by foxes and badgers. Details of the number and settlement of den complexes that were initially assessed in three regions of Poland are presented in Table 1.

Cubs were captured at 14 out of 33 (42.4%) sites where traps were set. At one site in the Czempiń area and three sites in the Białobrzegi area, trapping was conducted over two years (details in Table 2). At three sites, litters were relocated by the vixen after the successful trapping of a single cub. Two of these sites were located about 150 m from human settlements. The lowest number of trap-nights, during which cubs explored the inside of a trap, was recorded in the first distance class (≤ 150 m). Here, avoidance of traps was recorded two times more often than in the second distance class (151–300 m). In subsequent distance classes (2nd to 5th) willingness to explore and enter traps gradually increased (Table 3). The distance of a den to human settlements had a strong significant effect on the willingness of cubs to enter and explore traps (χ^2^ = 70.80, df = 5, *p* < 0.001).

During the three-year-period, trapping was carried out for 47 nights, involving 286 trap-nights (Table 2). As a result, 25 fox cubs (10 females and 15 males) were captured. The number of trapped cubs varied from 1 to 3 animals per site, however, we never managed to capture all the individuals from a single litter. At six sites (6/33; 18.2%) cubs were recaptured, including one site in the Czempiń area where one cub was recaptured twice, giving 32 trappings in total. The overall number of trapped and re-trapped animals in May and June was very similar (Table 4). Thus, there was no temporal difference in trappability of cubs. The sex of trapped animals was almost evenly distributed in both months of trappings (Table 4). The differences in sex distribution between May and June were not significant (*p* > 0.05). Males represented 67% (4/6 captures), 100% (8/8 captures), and 27% (3/11 captures) of captured foxes in 2016, 2017 and 2018 respectively.

Overall, the rate of successful trappings was 11.2 cubs/100 trap-nights. When cubs trapped only for the first time were considered, the trap rate was 8.7 cubs/100 trap-nights. The trap rate varied considerably between years of the study and was 5.5 cubs/100 trap-nights in 2016, 28.2 in 2017 and 12.9 in 2018. However, this result was not statistically comparable because the number of trap-nights was not equivalent between years. Nevertheless, the capture rate in the first year was the lowest in spite of the largest number of trap-nights (146 trap-nights in 2016 versus 39 in 2017 and 101 in 2018, Table 2). The probability of capturing one cub per night was high (70.2%; 32 cubs/47 nights). However, in the first year of the study this indicator was the lowest (44.4%; 8 cubs/18 nights). In the next two years, when an improved protocol was implemented, values of this indicator were comparable: 84.6% (11 cubs/13 nights) in 2017 and 81.3% (13 cubs/16 nights). The selectivity of the live-cage trapping method in this study was high (32/35 = 91.4%). Animals other than foxes were trapped only three times: a young badger, an Eurasian magpie (*Pica pica*) and a domestic dog (*Canis lupus familiaris*).

When human activity in the vicinity of the den site was considered, we found that twenty cubs (13.3 cubs/100 trap-nights) were captured when the trapping site was visited no more than two times before activation of a trap’s trigger mechanism. When the number of approaches was higher than two, 12 cubs (9.1 cubs/100 trap-nights) were trapped. This result was not significant (baiting × trapped: χ^2^ = 2.52, df = 1, *p* = 0.112). Nevertheless, the declining trend of successful capture with increased human activity in the vicinity of the burrow complex, suggests that human activity may have a negative effect on trapping success. Moreover, when the number of approaches was kept low, cubs explored the inside of traps more often (68.2%; 105/154 trap-nights) compared with sites visited more than twice (56.8%; 75/132 trap-nights). The statistically significant interaction of baiting x eagerness, describing this association, confirms this conclusion (χ^2^ = 3.934, df = 1, *p* = 0.047). 

The factor “weather” was positively associated with trapping success (χ^2^ = 20.40, df = 1, *p* < 0.001). Of 32 trapped animals, twelve animals (37.5%) were trapped during good weather conditions (5.8%; 12/207 trap-nights), while the remaining 20 cubs (62.5%) were captured when rain (n = 3), a thunderstorm (n = 15) or ground frost (n = 2) were recorded. 

## 4. Discussion

Safe and efficient trapping of red fox cubs is essential when invasive procedures on live animals need to be implemented and when subsequently these animals need to be released back into the wild (i.e., in scientific research, health surveillance, relocation or others). We have demonstrated here that our methodology of trapping red fox cubs with live-capture cage traps was effective, and that the captured animals did not suffer from any apparent injuries. The SOPs developed in this study seem to have had a positive impact on the behaviour of cubs (willingness of cubs to explore and/or enter the trap). However, only a complex behavioural study could verify this presumption. Nevertheless, we assessed the actual benefits resulting from the implementation of the improved SOPs based on efficiency of trappings and willingness of cubs to explore and enter traps. Importantly, we found that poor weather conditions can facilitate the success of trapping, while the presence of people at the trapping site and in close vicinity to den complexes may have a negative impact on the success of trapping.

One of the indicators used to assess the efficiency of our methodology was the trap rate. Values of this indicator were relatively high in comparison with other studies on juvenile canids. In the long-term study from Bristol, UK, the trap rate for juvenile red fox males and females was 4.20/100 trap-nights and 5.77/100 trap-nights, respectively [4]. A high trap rate (0.59 per trap-night) when using live-capture cage traps was found only when the method was modified [12]. In this approach, an improved box-trap enclosure was tested to capture kit fox (*Vulpes macrotis*) cubs in Utah, USA [12]. Frey et al. [6] suggested that specific environmental features of the study area can affect the trap rate when using live-capture cage traps. The effectiveness of this method was only 0.5/100 trap-nights in humid conditions and this was explained by the rapid rusting of cage material that was easily detectable by animals [6]. 

Assessment of the trap rate and the probability of capturing one cub per night in our study indicate that the experience of people involved in the field work contributed to the effectiveness of trapping. The relatively high probability of capturing one cub per night achieved in 2017 and 2018 is comparable with values of this indicator (83%) obtained in the study by Kozlowski et al. [12], but our results were much lower, half the value, in 2016. Likewise, the trap rate varied between years, and the value of this indicator in 2017 was about five times higher than in 2016 and two times higher than in 2018. In this context, it is important to mention that the protocol for trapping was improved based on our experience and observations in 2016 when we had initiated the field work (as described in the section entitled “Trappings of red fox cubs and safety measures”). The modifications implemented in our SOPs aimed to minimalize human presence at the site of trapping, after we had found that three litters of cubs were relocated by vixens as a result of unrest at the den site. Trapper experience was also considered to be a significant factor affecting capture rate by Ruette et al. [13]. These authors found a highly significant correlation between the trap rate and general experience of a trapper or experience of trapping at particular site. This was explained by a greater caution when setting and controlling traps, which is consistent with our observations. However, Ruette et al. [13] evaluated only foot snares and neck snares utilized for the capture of red foxes.

In our study, the human factor was also considered via another approach: distance of dens to areas inhabited and cultivated by people. Cubs inhabiting den complexes in the vicinity of human settlements avoided traps more frequently than cubs from other dens located more distantly. This result may indicate that animals living close to human settlements are more cautious and tend to avoid new elements in their territory. For example, at one site placed about 50 m from the local football field, a camera trap was installed for several days. Here, cubs were registered eating scraps of bait in the vicinity of traps, but they never approached or entered any of these cage traps. The same behaviour was observed at two sites that were surrounded by intensively cultivated arable land, where people were present on a daily basis.

No matter how willing the cubs were to explore a trap, we never managed to trap all the individuals from any given litter. We were unable to identify factors that prevented other individuals from the same litter from entering a trap. However, trap-aversion behaviour, manifested by avoidance of entering traps, has been described in other species of foxes [12,14,15]. Animals that had been captured once, developed this behaviour easily and quickly. Nevertheless, in a study conducted in Bristol, UK, out of 138 animals trapped as juveniles, 107 (78%) were trapped again within 4 months following the first trapping [4]. Similar to our study, the British group [4] did not find any sex differences in the trappability of both age groups: adult or juvenile foxes.

The willingness of cubs to explore and enter a trap was positively correlated with poor weather conditions. Interestingly, ten out of eleven cubs (including two recaptured animals) were trapped within one week in the Czempiń region in 2018. In the first weeks of trapping in this region, the weather was hot and dry. Heavy rains and thunderstorms occurred in this region only in the last week, when 10 cubs were captured. Thus, the lower trap rate in 2018 compared to that in 2017 could be explained by the weather conditions occurring during the course of our field work. There may be two explanations for the higher trap rate during poor weather conditions. First, rain helps to cover/remove any human scent in the vicinity of traps and den. On the other hand, when poor weather conditions impede hunting by the vixen, the cubs may be more eager to take the bait from traps. 

The selectivity of live-cage traps in our study was relatively high. However, our findings contrast with other studies comparing different devices utilized to capture adult foxes, and in which live-capture cage traps had the lowest selectivity. In a study from Argentina where the pampas fox (*Pseudalopex gymnocercus*) was a target, the selectivity of foot-hold traps, neck snares and live box traps was 61%, 100% and 32% respectively [16]. In a study performed on red foxes in Spain, the selectivity of neck snare traps (91.7%) and foot-hold traps (89.3%) was significantly higher than live cage traps (60%) [17]. However, these authors indicated different selectivity between sites with the highest variation for foot-hold traps (33.3–91.7%). Therefore, when considering the suitability of live-capture cage traps and their selectivity for a particular project in the future, environmental factors and the structure of the local wildlife community should be taken into account. However, live-capture cage traps have a few known disadvantages and thus in general this tool is used less willingly than other devices for capturing wild canids [17]. Preparations for trap setting are time consuming. The size of traps makes them difficult to set at some sites with dense vegetation or on uneven and hard surfaces. The traps are described as heavy and unhandy [12,17]. Malfunction of the trigger mechanism is another factor that can hinder trappings or even scare away the animals and cause trap-aversion [16].

## 5. Conclusions

The results of this study show that the use of live-capture cage-traps can be an effective, safe and selective method for red fox cubs. Animals reared in distant locations to human settlements are more likely to explore and enter the traps. Poor weather conditions and previous experience of the people engaged in a particular project involving field work may be considered as positive factors facilitating effective trapping.

## Figures and Tables

**Table 1:** Fox den complexes inspected during the three-year study. The table shows detailed information on the total number of dens inspected and inhabited by burrow dwelling animals in each of three regions where the study was conducted.

Number of Burrow Complexes (%)				
Location	settled/inspected	settled by fox cubs	settled by badgers	trapping attempted
Białobrzegi *	26/39 (66.7%)	20/39 (51.3%)	8/39 (20.5%)	18/39 (46.2%)
Czempiń *	23/40 (57.5%)	18/40 (45%)	8/40 (20%)	12/40 (30%)
Ujazd	4/18 (22.2%)	3/18 (16.7%)	1/18 (5.5%)	3/18 (16.7%)
TOTAL	53 */97 (54.6%)	41 */97 (42.3%)	17 */97 (17.5%)	33/97 (34%)

* Five den complexes were co-habited by fox cubs and badgers in the Białobrzegi (n = 2) and Czempiń (n = 3) areas.

**Table 2:** Summary of data on trappings at each region in the three-year study. ND = no data.

Region	Number of Den Complexes where Trappings were Attempted			Trap-Nights				Number of Trapped Cubs (Recaptures)			
	2016	2017	2018	2016	2017	2018	Total	2016	2017	2018	Total
Białobrzegi	10	7	4	146	13	21	180	6 (2)	2	2	10 (2)
Czempiń	ND	4	9	ND	23	70	93	ND	5 (3)	9 (2)	14 (5)
Ujazd	ND	1	2	ND	3	10	13	ND	1	ND	1
Total	10	12	15	146	39	101	286	6	8	11	25 (32)

**Table 3:** Willingness of cubs to enter and explore the trap expressed as the number of trap-nights when cubs entered the trap versus total number of trap-nights associated with a particular class of the distance from the den to the nearest village or farm. The ratio of number of cubs captured in six classes of distance to the total number of captured foxes is shown in the bottom row.

Distance Classes						
Class	1	2	3	4	5	6
	≤ 150 m	151–300 m	301–450 m	451–600 m	601–750 m	≥751 m
The number of cubs, which entered the traps	8/39	48/93	42/58	36/47	33/34	13/15
	20.5%	51.6%	72.4%	76.6%	97.1%	86.7%
No of trapped cubs	2/25 (8%)	5/25 (20%)	8/25 (32%)	6/25 (24%)	2/25 (8%)	2/25 (8%)

**Table 4:** Comparison of the number of captured red fox cubs in two months of trappings.

Trapped Red Fox Cubs		May	June
Number of trapped and re-trapped red fox cubs per trap-nights		15/111 (13.5%)	17/175 (9,7%)
Sex of trapped red fox cubs	male	7 (54.5%)	8 (61.5%)
	female	5 (45.5%)	5 (38.5%)

## References

[1] Iossa G., Soulsbury C.D., Harris S. (2007). Mammal trapping: a review of animal welfare standards of killing and restraining traps. Anim. Welfare-Potters Bar Then Wheathampstead..

[2] Marks C.A. (2010). Haematological and biochemical responses of red foxes (*Vulpes vulpes*) to different capture methods and shooting. Anim. Welf. (South Mimms, England).

[3] Sikes R.S., Gannon W.L. (2011). Guidelines of the American Society of Mammalogists for the use of wild mammals in research. J. Mammal..

[4] Baker P.J., Harris S., Robertson C.P.J., Saunders G., White P.C.L. (2001). Differences in the capture rate of cage-trapped foxes (*Vulpes vulpes*) and their implications for rabies contingency planning in Britain. J. Appl. Ecol..

[5] Iossa G., Soulsbury C.D., Baker P.J., Harris S. (2008). Body mass, territory size, and life-history tactics in a socially monogamous canid, the red fox. Vulpes vulpes. J. Mammal..

[6] Frey S.N., Conover M.R., Cook G. (2007). Successful use of neck snares to live-capture red foxes. Human-Wildlife Confl..

[7] Fleming P.J., Allen L.R., Berghout M.J., Meek P.D., Pavlov P.M., Stevens P., Kevin Strong K., Thompson J.A., Thomson P.C. (1998). The performance of wild-canid traps in Australia: efficiency, selectivity and trap-related injuries. Wildl. Res..

[8] Goszczyński J. (1995). Lis. Monografia przyrodniczo-łowiecka.

[9] Map Data ©2020 Google Google Maps. https://www.google.pl/maps.

[10] Behnke J.M., Bajer A., Harris P.D., Newington L., Pidgeon E., Rowlands G., Sheriff C., Kuliś-Malkowska K., Siński E., Gilbert F.S. (2008). Temporal and between-site variation in helminth communities of bank voles (*Myodes glareolus*) from NE Poland. 1. Regional fauna and component community levels. Parasitol..

[11] Grzybek M., Bajer A., Bednarska M., Al-Sarraf M., Behnke-Borowczyk J., Harris P.D., Price S.J., Brown G.S., Osborne S.-J., Siński E. (2015). Long-term spatiotemporal stability and dynamic changes in helminth infracommunities of bank voles (*Myodes glareolus*) in NE Poland. Parasitol..

[12] Kozlowski A.J., Bennett T.J., Gese E.M., Arjo W.M. (2003). Live capture of denning mammals using an improved box-trap enclosure: kit foxes as a test case. Wildl. Soc. Bull..

[13] Ruette S., Stahl P., Albaret M. (2003). Factors affecting trapping success of red fox *Vulpes vulpes*, stone marten *Martes foina* and pine marten *M. martes* in France. Wildl. Biol..

[14] Cypher B.L., Warrick G.D., Otten M.R., O’Farrell T.P., Berry W.H., Harris C.E., Kato T.T., McCue P.M., Scrivner J.H., Zoellick B.W. (2000). Population dynamics of San Joaquin kit foxes at the Naval Petroleum Reserves in California. Wildl. Mono..

[15] Schauster E.R., Gese E.M., Kitchen A.M. (2002). An evaluation of survey methods for monitoring swift fox abundance. Wildl. Soc. Bull..

[16] Vidal E.M.L., Lucherini M., Casanave E.B. (2003). An evaluation of three restraining devices for capturing pampas foxes. Canid News..

[17] Muñoz-Igualada J.A.I.M.E., Shivik J.A., Domínguez F.G., Lara J., González L.M. (2008). Evaluation of cage-traps and cable restraint devices to capture red foxes in Spain. J. Wildl. Manag..

